# Robust Immunity and Heterologous Protection against Influenza in Mice Elicited by a Novel Recombinant NP-M2e Fusion Protein Expressed in *E. coli*

**DOI:** 10.1371/journal.pone.0052488

**Published:** 2012-12-21

**Authors:** Wenling Wang, Baoying Huang, Tao Jiang, Xiuping Wang, Xiangrong Qi, Yingying Gao, Wenjie Tan, Li Ruan

**Affiliations:** National Institute for Viral Disease Control & Prevention, Chinese Center for Disease Control and Prevention, China CDC, Beijing, People’s Republic of China; The Ohio State University, United States of America

## Abstract

**Background:**

The 23-amino acid extracellular domain of matrix 2 protein (M2e) and the internal nucleoprotein (NP) of influenza are highly conserved among viruses and thus are promising candidate antigens for the development of a universal influenza vaccine. Various M2e- or NP-based DNA or viral vector vaccines have been shown to have high immunogenicity; however, high cost, complicated immunization procedures, and vector-specific antibody responses have restricted their applications. Immunization with an NP–M2e fusion protein expressed in *Escherichia coli* may represent an alternative strategy for the development of a universal influenza vaccine.

**Methodology/Principal Findings:**

cDNA encoding M2e was fused to the 3′ end of NP cDNA from influenza virus A/Beijing/30/95 (H3N2). The fusion protein (NM2e) was expressed in E. coli and isolated with 90% purity. Mice were immunized with recombinant NM2e protein along with aluminum hydroxide gel and/or CpG as adjuvant. NM2e plus aluminum hydroxide gel almost completely protected the mice against a lethal (20 LD_50_) challenge of heterologous influenza virus A/PR/8/34.

**Conclusions/Significance:**

The NM2e fusion protein expressed in *E. coli* was highly immunogenic in mice. Immunization with NM2e formulated with aluminum hydroxide gel protected mice against a lethal dose of a heterologous influenza virus. Vaccination with recombinant NM2e fusion protein is a promising strategy for the development of a universal influenza vaccine.

## Introduction

Currently, vaccination is the most effective method for prevention of influenza [1], [2]. However, conventional flu vaccines based on hemagglutinin (HA) and neuraminidase (NA) have failed to induce heterosubtypic protection owing to the high variability of these two antigens [3]–[5]. To afford intrasubtypic and heterosubtypic cross-protection, a universal influenza vaccine based on the more conserved antigens of influenza viruses is desirable, as conserved antigens are consistent across strains and do not exhibit frequent variation [2], [6], [7]. Matrix 2 protein (M2) and nucleoprotein (NP) are conserved antigens of influenza A virus and thus are promising candidate antigens for the development of a universal influenza vaccine [8], [9]. Recent studies have investigated the potential of M2 (mainly M2e) [10]–[19] or NP [20]–[24] as alternative antigens in preventing seasonal and pandemic flu outbreaks. In these cases, M2e was fused genetically or linked chemically with large carriers such as hepatitis B virus core (HBVc), flagellin, phage Qβ, *Neisseria meningitides* outer membrane complex (OMPC). M2 and NP have also been used together, because the combination of multiple antigens is often superior to a single antigen in terms of eliciting an immune response. In previous studies, injections of vaccines based on NP and M2 recombinant DNA and/or adenovirus have conferred protection to mice against a lethal virus challenge, and it showed that the protection induced by the combination of NP and M2 was superior to the sole one [25]–[31]. However, the poor immunogenicity of DNA-based vaccines may restrict their wide application [32], and vector-based vaccines have the potential to elicit anti-vector antibodies which may interfere with immunization [33].

A prokaryotic system may be the simplest and fastest method for expression and purification of large quantities of a single antigenic protein for the production of a new influenza vaccine [34], [35]. The influenza A virus NP protein [36] and M2 protein with residues 26–55 deleted [37] have been expressed fom *E. coli* successfully and they induced broad protective immunity against influenza. A fusion protein consisting of NP and M2 expressed in a prokaryotic system is a promising candidate antigen for a universal influenza vaccine, and the new construct may contain the character of the two antigens. Protein vaccines are superior to attenuated live vaccines and inactivated virus vaccines with respect to safety. However, due to the poor immunogenicity of protein vaccines, an appropriate adjuvant must be used to induce effective and long-term protection [38]. The development of more effective vaccine and adjuvant formulations, as well as procedures to enhance the immunogenicity of influenza virus proteins and peptides, could result in improved humoral and cell-mediated immunity [15], [39]–[43].

In this study, NP and the 23-amino acid extracellular domain of M2 (M2e), which are highly conserved among viruses, were selected as candidate antigens for a universal influenza A vaccine. The cDNA encoding M2e was fused to the 3′ end of the full-length cDNA for NP from influenza virus A/Beijing/30/95 (H3N2). The resultant fusion protein (NM2e) was expressed in *Escherichia coli* and isolated with 90% purity. Recombinant NM2e fusion protein was highly immunogenic in mice, and NM2e formulated with aluminum hydroxide gel as an adjuvant almost completely protected the mice against challenge with a lethal dose of heterologous influenza virus A/Puerto Rico/8/34. Thus, NM2e fusion protein expressed in *E. coli* is a promising candidate antigen for the development of a universal influenza vaccine.

## Materials and Methods

### Ethics Statement

This mouse study was conducted in strict accordance with the recommendations in the Guide for the Care and Use of Laboratory Animals of the Chinese Center for Disease control and prevention. The protocol was approved by the Committee on the Ethics of Animal Experiments of the Institute for Occupational Health and Poison Control (Permit Number: EAWE-2010-029). Serum was obtained by orbital sinus puncture. In the ELISPOT assay mice were sacrificed by cervical dislocation. Challenge experiment was performed under sodium pentobarbital anesthesia, and all efforts were made to minimize suffering. After influenza a virus PR8 challenge, mice were monitored closely for approximately 21 days for signs of illness. Any animals in a moribund condition were euthanized.

### Construction, Expression, and Purification of NM2e Fusion Protein

**Figure 1:**
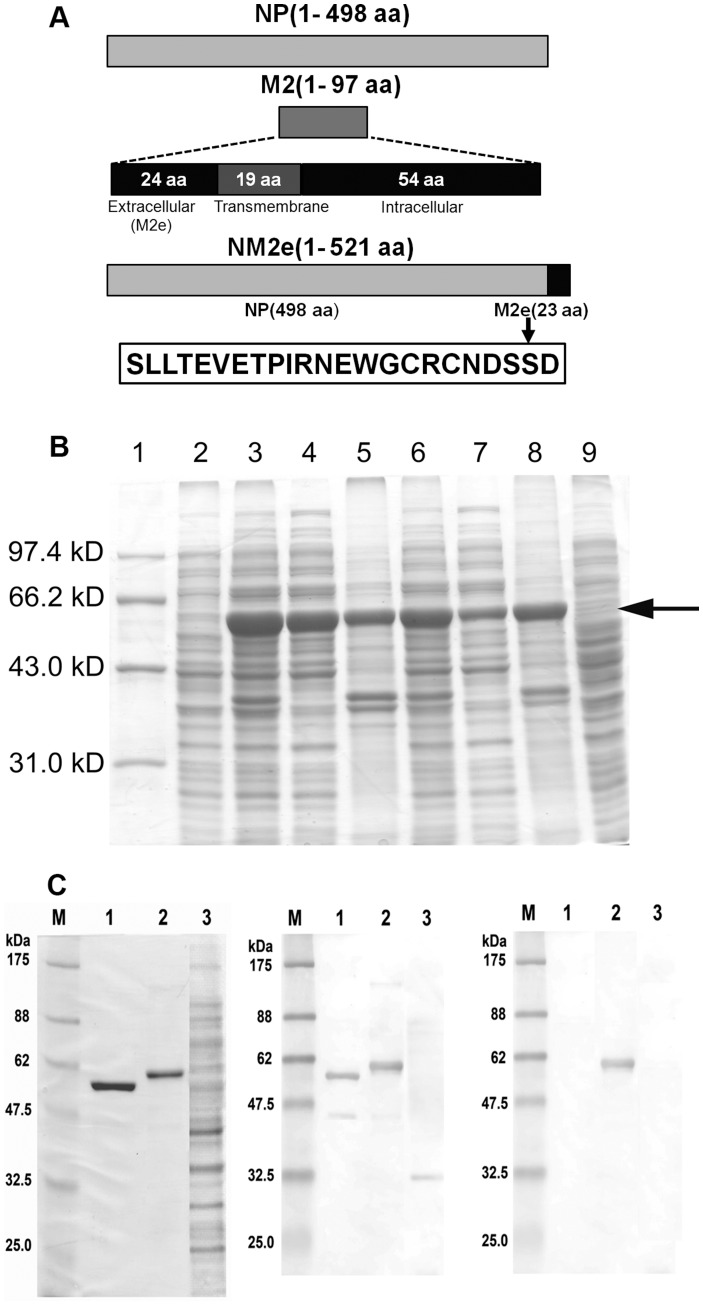
Construction, expression, and characterization of NM2e fusion protein. (A) Schematic of influenza A virus NP and M2, and diagram for the construction of recombinant NM2e fusion protein. The entire sequence of NM2e is shown. The cDNA sequences encoding residues 1–498 of NP and residues 2–24 of M2 (extracellular domain of M2, M2e) from influenza A virus A/Beijing/30/95 (H3N2) were fused directly, with no linker, and this was cloned as pET30a-NM2e for expression in *Escherichia coli*. (B) Protein profile of cell lysates from induction experiments in *E. coli* BL21 (DE3) transformed with pET30a-NM2e at 25 and 37°C. Lane 1, Mid-range protein molecular weight marker; lane 2, whole-cell lysate of transformed *E. coli* before induction; lanes 3–5, whole-cell lysate, soluble supernatant, and insoluble fraction after 4-h induction with 0.1 mM IPTG at 25°C; lanes 6–8, whole-cell lysate, soluble supernatant, and insoluble fraction after 4-h induction with 0.1 mM IPTG at 37°C; lane 9, whole-cell lysate after 4 h of cultivation without IPTG at 37°C. The arrow indicates the 58-kDa band corresponding to NM2e. (C) SDS-PAGE (left) showing the purified NM2e fusion protein, NP of influenza A virus A/Beijing/30/95(H3N2), and lysates of *E. coli* transformed with pET30a(+). NM2e fusion protein was detected on Western blots probed with NP-immunized mouse serum (middle) and mouse anti-M2e monoclonal antibody (right). Lanes 1–3, purified influenza A virus NP, recombinant NM2e fusion protein expressed in *E. coli,* cell lysates of *E. coli* transformed with pET30a(+). M, protein molecular weight marker.

Genes of influenza A virus A/Beijing/30/95 (H3N2) strain (supplied by Department of Influenza Virus, National Institute for Viral Disease Control and Prevention, China CDC) were used as templates for cloning NP and M2e. The cDNA encoding M2e (amino acid sequence: SLLTEVETPIRNEWGCRCNDSSD) was fused to the 3′ end of the full-length cDNA for NP (498 amino acids) without any linker sequence to form the fusion construct NM2e (Fig. 1). The optimized NM2e cDNA was synthesized and ligated into the pET-30a(+) vector (Merck-Novagen, Darmstadt, Germany) between the *Nde*I and *Eco*RI sites to form pET30a-NM2e, which was used to transform *E. coli* BL21(DE3) (Merck-Novagen). The expressed NM2e protein was purified by ion exchange chromatography (DEAE-Sepharose Fast Flow, Amersham Biosciences Corp., New Jersey, USA), followed by gel chromatography (Superdex 200, Amersham Biosciences Corp, New Jersey, USA). The purified NM2e was concentrated, quantified using the bicinchonic acid protein assay (PIERCE Biotechnology, Rockford, Illinois, USA), and stored at −70°C. Endotoxin levels were determined using the Tachypleas Amebocyte Lysate assay (Chinese Horseshoe Crab Reagen Manufactory, Xiamen, China) as directed by the manufacturer. The endotoxin level of the NM2e protein was about 2000 EU/mg. Furthermore, the recombinant NM2e was analyzed by Western blot assay. Samples were electrophoresed in a 12% polyacrylamide gel under reducing conditions, transferred to nitrocellulose, probed with influenza A virus NP-immunized murine serum and a monoclonal antibody (14C2**;** Abcam, Cambridge, UK) against influenza A virus M2e, and incubated with HRP-conjugated goat anti-mouse IgG (1∶10000) (Sigma-Aldrich, St. Louis, Mousa, USA). Immunoreactivity was detected with 3, 3′-diaminobenzidine (DAB) substrate.

### Immunogenicity and Protective Efficacy of NM2e Immunization in mice

**Figure 2:**
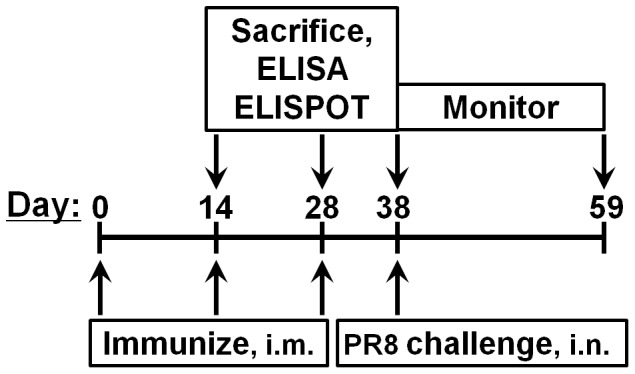
NM2e protein immunization schedule. The indicated mice were immunized intramuscularly with NM2e protein with or without adjuvant, three times at 2-week intervals. Blood was collected on days 14, 28, and 38, respectively. The immunized mice were challenged with influenza A virus PR8 at 20-fold the LD_50_ on day 38. Body weight and survival were monitored for 3 weeks, until day 59.

The immunogenic potential of NM2e fusion protein was evaluated in six groups of 4- to 6-week-old BALB/c (H-2^d^) mice. Mice were purchased from the Institute of Laboratory Animals, Chinese Academy of Medical Sciences, and raised in cages in the Institute for Occupational Health and Poison Control, Chinese center for Disease Control and Prevention. Aluminum hydroxide gel (Al[OH]_3_, Alhydrogel; Brenntag Biosector, DK-3600 Frederikssund, Denmark) and/or CpG 1826 (5′-TCCATGACGTTCCTGACGTT-3′) were used as adjuvants. The mice in each group (*n* = 33) were injected intramuscularly in the right posterior gastrocnemius as follows: group 1, 0.9% NaCl solution (normal saline, NS); group 2, NS plus Al(OH)_3_ and CpG 1826; group 3, 10 µg of NM2e; group 4, 10 µg of NM2e plus CpG 1826; group 5, 10 µg of NM2e plus Al(OH)_3_; and group 6, 10 µg NM2e plus Al(OH)_3_ and CpG 1826 (Table 1). Immunization was performed three times at 2-week intervals. Blood samples were collected by orbital sinus puncture from six mice in each group at the times indicated in Fig. 2, and mice were sacrificed by cervical dislocation, then spleen were removed and grinded up sterilely. Spleen mononuclear cells (SMNCs) were obtained after the red blood cells in the spleen cell suspension were lysed. Ten days after the third immunization, the protective potential of recombinant NM2e fusion protein was evaluated in the vaccinated mice. Mice were anesthetized intraperitoneally with sodium pentobarbital (10 mg/ml) at a dose of 60 mg/kg body weight and infected with 50 µl of influenza A/PR/8/34 (H1N1) (abbreviated PR8), containing 20-fold the LD_50_. The daily body weight loss and mortality were monitored for 3 weeks after the challenge.

**Table 1:** Summary of mouse groups immunized with NM2e.

Group	NM2e/dose^a^	Adjuvant/dose^b^	Volume
G1	(NS/triple dose)	–	100 µl
G2	(NS/triple dose)	Al(OH)_3_/100 µg+CpG/10 µg	100 µl
G3	Triple dose/10 µg	–	100 µl
G4	Triple dose/10 µg	CpG/10 µg	100 µl
G5	Triple dose/10 µg	Al(OH)_3_/100 µg	100 µl
G6	Triple dose/10 µg	Al(OH)_3_/100 µg+CpG/10 µg	100 µl

NS, normal saline.

a mice in G1 and G2 were mock-immunized with NS instead of NM2e protein.

b mice in G1 and G3 were immunized without adjuvant.

### ELISA Protocol

Specific antibodies from serum samples of NM2e-immunized mice were determined by ELISA. Microtiter plates were coated with 2 µg/ml recombinant NP [44] or M2e peptide (synthesized in Beijing Scilight Biotechnology Ltd. Co., Beijing, China) overnight at 4°C. Washed wells were blocked by incubation with PBS containing 2% BSA (Amresco, Solon, Ohio, USA) for 2 h at 37°C. Serum samples were serially diluted in PBS containing 1% BSA, added to the wells, and incubated for 1.5 h at 37°C. After repeated washes, bound total IgG and IgG1 and IgG2a isotypes were detected by incubation with HRP-conjugated goat anti-mouse IgG (Sigma) (1∶10000 dilution), IgG1 (Southern Biotech, Birmingham, Alabama, USA) (1∶5000 dilution), and IgG2a (Southern Biotech) (1∶5000 dilution), respectively, for 1.5 h at 37°C. After washing, 100 µl of TMB substrate solution was added to each well, and plates were incubated for 5 min at room temperature in darkness. The reaction was stopped by addition of 50 µl of 1 M H_2_SO_4_ to each well. The color produced by the enzymatic reaction was determined by measuring the absorbance at 450 nm.

### ELISPOT Protocol

Murine ELISPOT kits (BD Biosciences, Franklin Lakes, New Jersey, USA) were used to detect the numbers of IFN-γ-, IL-4-, and IL-10-secreting SMNCs separated from blood samples of mice immunized with NM2e protein. ELISPOT plates were coated with anti-murine IFN-γ, IL-4, and IL-10 antibodies overnight at 4°C. RPMI-1640 medium containing 10% FBS (R-10; GIBCO, Langley, Oklahoma, USA) was added to block nonspecific sites for 2 h at room temperature. SMNCs were aseptically isolated, and 5 × 10^5^ SMNCs suspended in 100 µl of R-10 containing 10 µg/ml NP_55–69_ (RLIQNSLTIERMVLS; H-2d-restricted Th epitope), NP_147–155_ (TYQRTRALV; H-2d-restricted CTL epitope), or M2e peptide pool (peptides of residues 1–15, 5–19, and 9–23) were added to each well. After incubation for 40 h in a 5% CO_2_ incubator at 37°C, 100 µl of detection antibody was added to each well and incubated for 2 h at room temperature. Then, 100 µl of enzyme complex solution was added, followed by incubation for 1 h at room temperature. AEC substrate solution (100 µl) was added to each well and the reaction was allowed to proceed for 20 min at room temperature in darkness. To terminate the reaction, the ELISPOT plate was rinsed with flowing water. An ELISPOT image analyzer (Bioreader 4000; Bio-Sys, Karben, Germany) was used to determine the number of spot-forming cells (SFCs).

### Statistical Analysis

Statistical analysis was performed using SPSS, version 17.0, and Prism, version 5.0a, software. Log conversion was performed for antibody titers. Differences in antibody titers and ELISPOT results among groups were analyzed by one-way ANOVA. The paired *t*-test and log-rank test were used to analyze differences in body weight change curves and survival rate curves, respectively. Differences with *p*-values ≤0.05 were considered significant.

## Results

### Design, Expression, Purification, and Identification of NM2e Fusion Protein

The constructed cDNA encoding a fusion protein containing full-length NP (498 amino acids) with the extracellular domain of M2 (M2e, 23 amino acids) at its C-terminus, with no linker sequence, was expressed in *E. coli* as a protein of 521 amino acids, and designated NM2e (Fig. 1A). Native sequences from influenza A virus A/Beijing/30/95 (H3N2) were used as templates for cloning the target genes. The codons of the NM2e cDNA sequence were optimized for expression in *E. coli*, and the synthesized cDNA was cloned in the prokaryotic expression vector pET-30a(+). The protein was expressed in *E. coli* BL21(DE3) with high efficiency (Fig. 1B). Most of the recombinant protein was produced as insoluble inclusion bodies with induction at 37°C, but as soluble protein with induction at 25°C. The optimal conditions for producing the maximal amount of soluble NM2e protein were induction with 0.1 mM IPTG for 12 h at 25°C. Under these culture conditions, the target protein accounted for 20–30% of the total soluble *E. coli* protein. The NM2e protein yield of the gene-codon optimized cDNA was two- to three-fold that of the original cDNA (data not presented).

The fusion protein was purified by ion-exchange chromatography followed by gel filtration chromatography, with a yield of 3.20 mg of NM2e protein per liter of culture medium. SDS-PAGE with Coomassie Blue staining showed that the fusion protein was 58 kDa and about 90% pure (Fig. 1C). On Western blots, the expressed NM2e protein was recognized by serum from NP-immunized BALB/c mice and by a monoclonal antibody against M2e (Fig. 1C). These results demonstrate the successful *in vitro* expression of NM2e protein and its isolation with 90% purity.

### An Immune Response in Mice is Elicited by NM2e Immunization

To determine the immunogenicity of recombinant NM2e protein, it was used to immunize groups of BALB/c mice three times at 2-week intervals, as described in Fig. 2. Immunogen and adjuvant doses used in each group are presented in Table 1.

**Figure 3:**
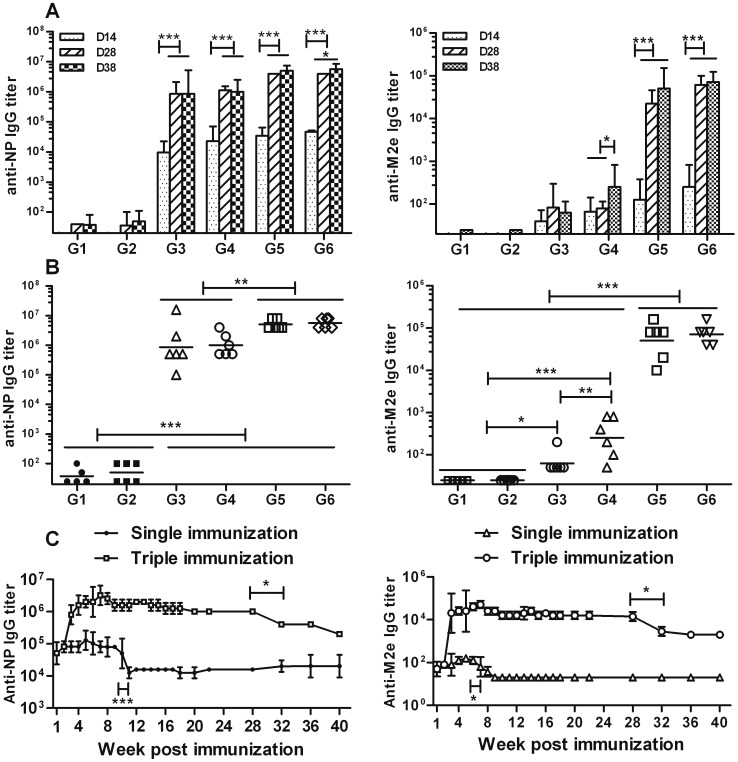
Antibody response trend and long-term humoral immune response induced by NM2e protein in mice. (A and B) Mice were immunized intramuscularly with 10 µg of NM2e protein three times at 2-week intervals. Al(OH)_3_ and/or CpG 1826 were used as adjuvants. Mice immunized with normal saline (NS) or adjuvant alone was used as negative controls. Serum was obtained from each mouse on days 14, 28, and 38, respectively, and analyzed for the presence of IgG antibodies specific for NP (left) or M2e (right), in an ELISA, as described in the Materials and Methods. Antibody response trends after three immunizations are presented in A, and the comparison of results on day 38 are presented in B. Columns show geometric mean antibody titers, and bars indicate the 95% confidence interval in each group. Plots in B show the NP- and M2e-specific IgG titers of all of the mice in each treatment group on day 38, and bars indicate the geometric mean antibody titers of each treatment group (*n* = 6 mice per experimental group, except *n* = 5 mice in the NS group). Lines above two or more groups indicate that they have the same comparative results. *, *p*≤0.05; **, *p*≤0.01; ***, *p*≤0.001 by one-way ANOVA. (C) Mice were immunized intramuscularly with 10 µg of NM2e protein formulated with Al(OH)_3_ three times at 2-week intervals or immunized with a single dose of 10 µg of NM2e formulated with Al(OH)_3_. Serum was prepared from each mouse at the indicated times, and NP- and M2e-specific IgG antibodies were analyzed by ELISA, as described in the Materials and Methods.

In group 3, the priming vaccination with NM2e alone induced a high titer of anti-NP IgG (9.6 × 10^3^) (Fig. 3A, left), but a low titer of anti-M2e IgG (40) (Fig. 3A, right). The second immunization significantly improved the serum anti-NP IgG titer (8.5 × 10^5^; *p*<0.001), but not that of anti-M2e IgG (82; *p*>0.05). The third immunization had no additional effect on either titer.

**Figure 4:**
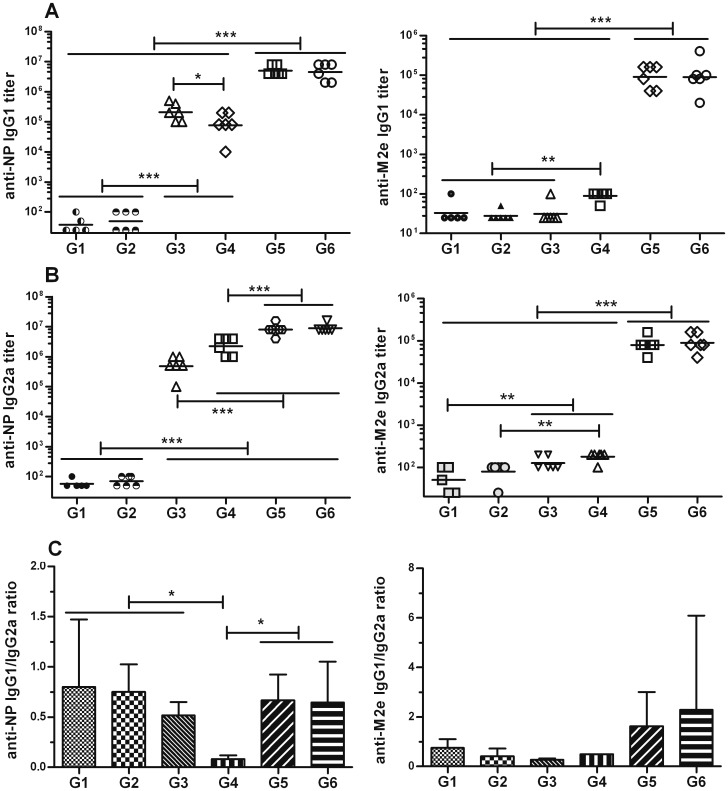
IgG1 and IgG2a isotypes in serum from NM2e-immunized mice. Mice were treated as described in Fig. 3. NP- and M2e-specific IgG isotypes in mouse serum were analyzed by ELISA. The plots show the (A) NP- (left) and M2e-specific (right) IgG1 isotypes and (B) the respective IgG2a isotypes. The scatter dot plots show the results for every mouse in each group, and the bars show the geometric mean of each group. The plots in (C) present the NP-(left) and M2e-specific (right) IgG1/IgG2a ratios, and the bars show the means with SD. Lines above two or more groups indicate that they have the same comparative results. *, *p*≤0.05; **, *p*≤0.01; ***, *p*≤0.001 by one-way ANOVA.

**Figure 5:**
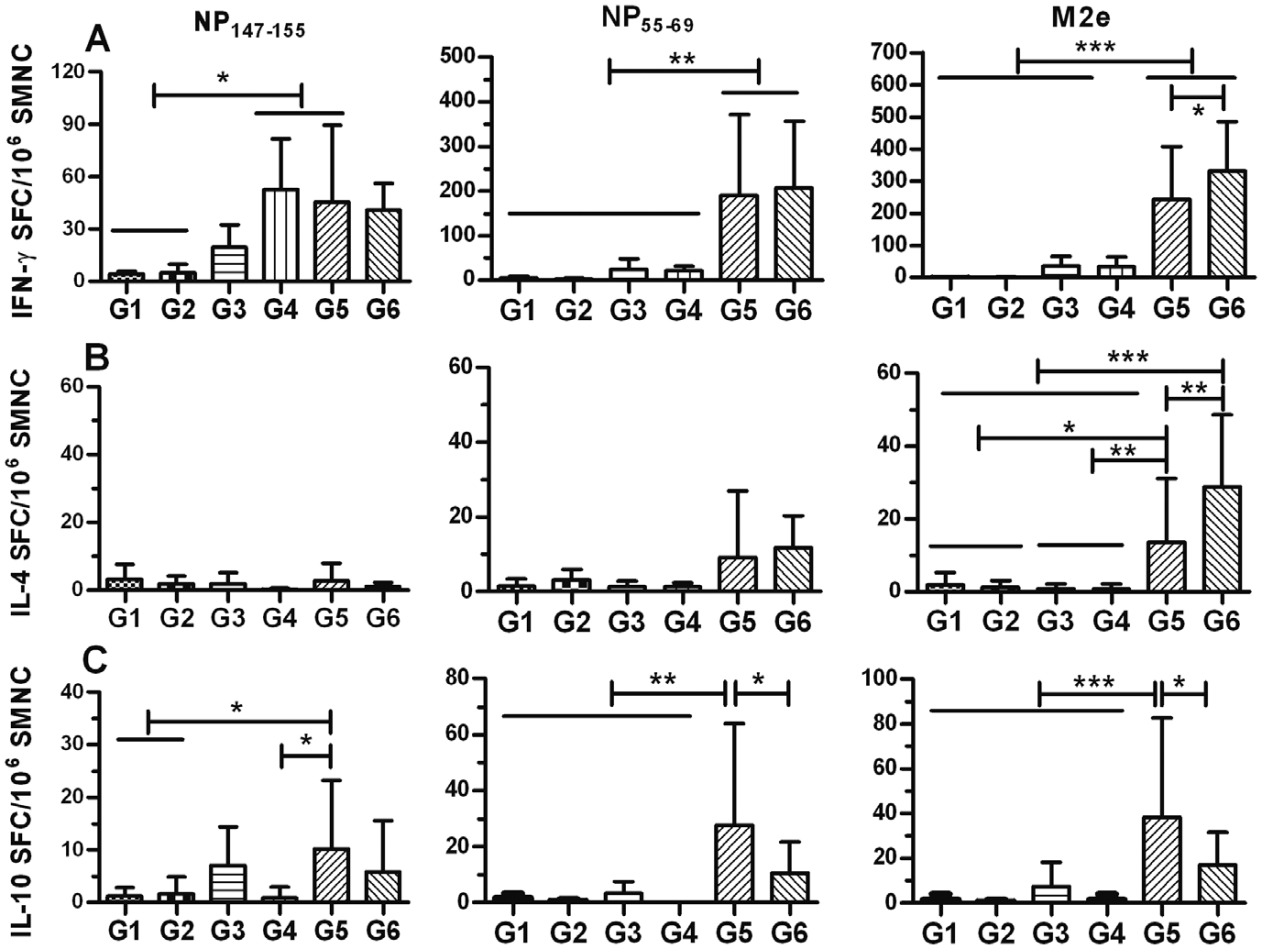
Cellular immune response in NM2e-immunized mice. SMNCs secreting IFN-γ, IL-4, or IL-10 upon stimulation were detected by ELISPOT assay. Groups of six mice were immunized intramuscularly three times at 2-week intervals using 10 µg of NM2e protein in normal saline (NS) or 10 µg of NM2e formulated with Al(OH)_3_, CpG 1826, or Al(OH)_3_ plus CpG 1826. Mice immunized with NS or Al(OH)_3_ plus CpG 1826 were treated as negative controls. All mice in each treatment group were sacrificed on day 38. SMNCs were separated from mouse spleen samples, and 5 µg/ml NP_147–155_, NP_55–69_, and M2e peptide pool were used as stimulants in the ELISPOT assays. The numbers of SMNCs producing IFN-γ (A), IL-4 (B), or IL-10 (C) after stimulation for 40 h with NP_147–155_ (left), NP_55–69_ (middle), or M2e peptides (right) are presented as spot-forming cells (SFCs)/10^6^ SMNCs. Columns show the average SFCs/10^6^ SMNCs, and bars indicate the standard deviation of each group. Lines above two or more groups indicate that they have the same comparative results. *, *p*≤0.05; **, *p*≤0.01; ***, *p*≤0.001 by one-way ANOVA.

To test whether an adjuvant could increase the immunogenicity of NM2e, the protein was formulated with aluminum hydroxide gel (Al[OH]_3_) alone (group 5). The inclusion of Al(OH)_3_ with NM2e induced higher levels of anti-NP IgG (3.4 × 10^4^) and anti-M2e IgG (126) after the prime immunization, which was significantly improved after the second immunization (4 × 10^6^, *p*<0.001 and 2 × 10^4^, *p*<0.001) (Fig. 3A). Based on IgG titers after the third immunization, the inclusion of Al(OH)_3_ significantly improved the anti-M2e (5 × 10^4^, *p*<0.001) and anti-NP IgG (5×10^6^, *p*<0.01) titers compared with immunization using NM2e protein alone (Fig. 3B). Long term monitoring data showed that when mice were immunized with NM2e formulated with Al(OH)_3_ three times at 2-week intervals, the high anti-NP and anti-M2e IgG levels were maintained for at least 7 months, while single immunization with the same formulation induced only low-level anti-NP and anti-M2e IgG, which was reduced further 3 months after the immunization (Fig. 3C). We can infer from the long-term data that NM2e formulated with Al(OH)_3_ induces stronger and longer-lasting anti-NP and anti-M2e IgG responses than does NM2e protein alone. Furthermore, we identified the anti-NP and anti-M2e antibody isotypes by ELISA (Fig. 4). NM2e immunization elicited high levels of anti-NP IgG1 (2 × 10^5^) and IgG2a (4 × 10^5^) (Fig. 4, left) after three immunizations. When Al(OH)_3_ was included in the formulation with NM2e, both the anti-NP and anti-M2e IgG1 and IgG2a titers were markedly increased (Fig. 4 A, B). To further characterize the cellular immune responses elicited by NM2e protein in mice, IFN-γ-, IL-4-, and IL-10-secreting SMNCs were quantified by ELISPOT assays (Fig. 5). The priming and second immunizations elicited only a few IFN-γ-, IL-4-, or IL-10-secreting SMNCs (data not shown), and the third immunization elicited a limited number of IL-4- or IL-10-secreting SMNCs (Fig. 5B, C) in each group. Therefore, only the IFN-γ-specific ELISPOT results were analyzed in detail. Immunization with NM2e alone did not elicit a clear cellular response, however, Al(OH)_3_ significantly enhanced the NM2e-induced cellular immune response when NP55-69 (*p*<0.01) (Fig. 5A, middle) and the M2e peptide pool (*p*<0.001) (Fig. 5A, right) were used as stimuli.

CpG oligodeoxynucleotide alone was used to increase the immunogenicity of NM2e in group 4 mice. The data showed that CpG improved the immune response elicited by NM2e, although it displayed poorer efficacy than Al(OH)_3_. The inclusion of CpG with NM2e induced higher levels of anti-NP IgG titer (2.3 × 10^4^) and low level of anti-M2e IgG titer (66) after the prime immunization, the anti-NP IgG was significantly improved after the second immunization (1.1 × 10^6^, *p*<0.001) (Fig. 3A, left), whereas the anti-M2e IgG titer was improved markedly till the third immunization (252, *p*<0.05) (Fig. 3A, right). Based on IgG titers after the third immunization, the inclusion of CpG significantly improved the anti-M2e IgG titer (252, *p*<0.01), but not the anti-NP IgG titer (2.3 × 10^4^, *p*>0.05) compared with immunization using NM2e protein alone (Fig. 3A, B, right). The anti-NP and anti-M2e IgG titers were significantly lower in group 4 than in group 5 (*p*<0.01 and *p*<0.001, respectively) (Fig. 3B). When antibody subtype was considered, the inclusion of CpG in NM2e significantly increased the NP-specific IgG2a titer (2 × 10^6^, *p*<0.001) and decreased the IgG1 titer (7 × 10^4^, *p*<0.05) compared with NM2e immunization alone. This resulted in a lower anti-NP IgG1/IgG2a ratio indicative of a potent Th1 response, which is different from the IgG1/IgG2a pattern in groups 3 and 5 (Fig. 4, left). Anti-M2e antibody subtype data showed that inclusion of CpG with NM2e improved the anti-M2e IgG1 titer (89), but not the IgG2a titer (178) (Fig. 4, right). Meanwhile, ELISPOT results suggested that the inclusion of CpG had no clear effect on the cellular immune response induced by NM2e alone (Fig. 5A).

Al(OH)_3_ and CpG were also used together to improve the immunogenicity of NM2e protein (group 6). Based on IgG titers after the third immunization, anti-NP and anti-M2e IgG titers, regardless of antibody subtype, of group 6 were not markedly higher than those of group 5 (Fig 3, 4). Meanwhile, the inclusion of Al(OH)_3_ plus CpG with NM2e failed to show a stronger cellular immune response than Al(OH)_3_ alone (Fig. 5).

These results indicate that the NM2e fusion protein expressed in *E. coli* is immunogenic in mice. Al(OH)_3_ markedly improved the immune response of NM2e in mice. However, NM2e formulated with CpG induced poorer immune responses than with Al(OH)_3_, although CpG also improved the immunogenicity of NM2e. Moreover, Al(OH)_3_ and CpG showed no clear synergistic effect when combined in the NM2e formulation.

### NM2e Induces Protection against Influenza A virus PR8 Challenge in Mice

**Figure 6:**
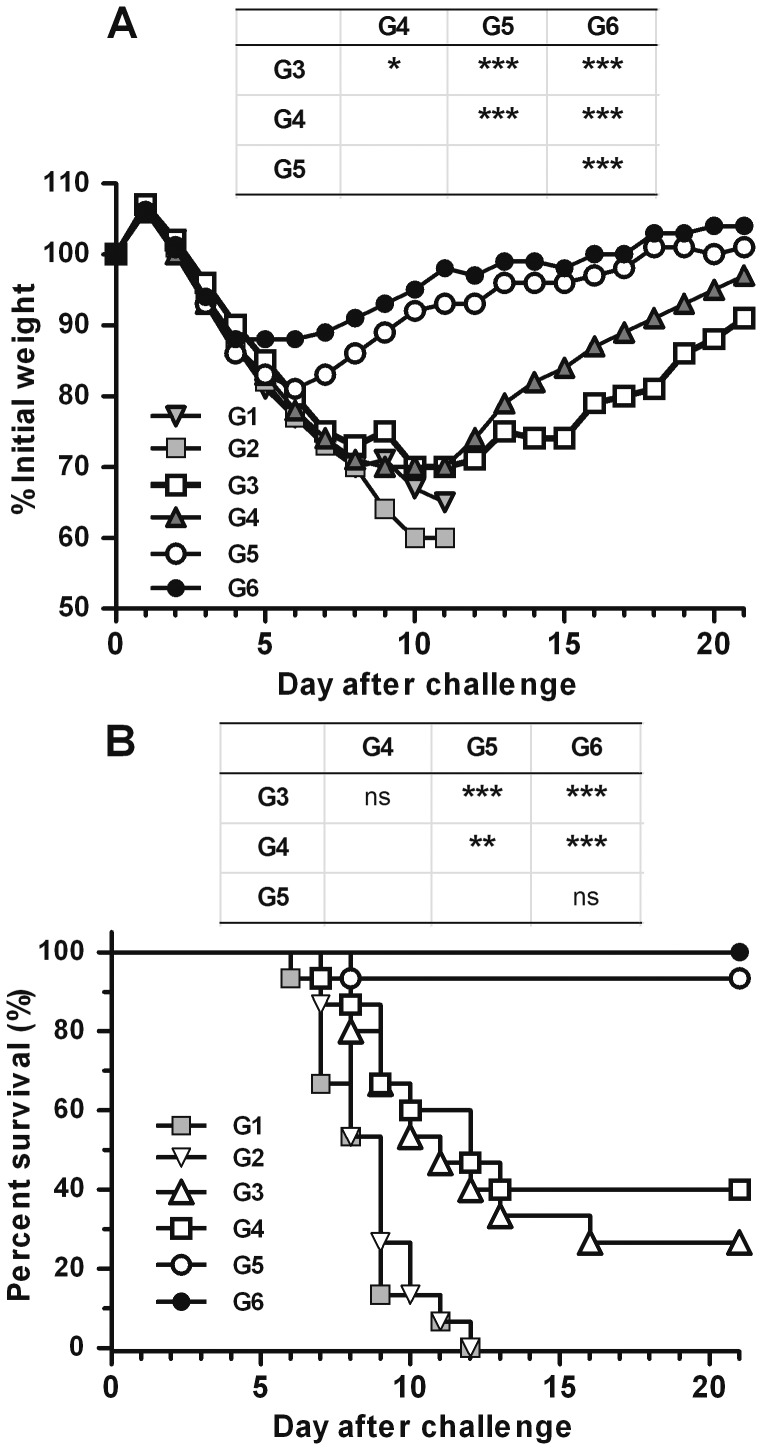
Protective efficacy of immunization with NM2e formulated with Al(OH)_3_ and CpG in mice. Groups of 15 mice were immunized with NM2e protein or NM2e formulated with adjuvant and were challenged with 20 LD_50_ of influenza virus PR8. Mice immunized with normal saline or adjuvant alone were challenged as negative controls. Mice were monitored daily for 21 days after PR8 challenge. Mice were weighed daily to detect morbidity (A). Average weights in each treatment group were followed for the duration of the study, and the percentage of the original body weight was calculated based on the average starting weight for each group at day 0. Survival rates (B) following the challenge within each experimental group were calculated. Tables above the graph compare the results for groups 3, 4, 5, and 6. *, *p*≤0.05; **, *p*≤0.01; ***, *p*≤0.001; ns, not significant.

We investigated the potential for immunization with the NM2e fusion protein to induce cross protection. All mice in control groups 1 and 2 showed marked weight loss and died from lethal infection upon heterologous virus challenge (Fig. 6). Mice immunized with NM2e alone showed severe body weight loss, as much as 30%, and only 27% of the mice survived the lethal virus challenge. Although weight loss was similar between the NM2e-only (group 3) and NM2e/CpG (group 4) immunized groups, 40% of the NM2e/CpG immunized mice survived the lethal challenge. Compared with groups 3 and 4, mice immunized with NM2e/Al(OH)_3_ (group 5) exhibited significantly lesser weight loss (19%, *p*<0.001) and significantly higher survival (93%, *p*<0.001). Although the transient weight loss in mice immunized with NM2e formulated with Al(OH)_3_ plus CpG (group 6) was slight (12%), the survival percentage was not significantly different from that of group 5 (*p*>0.05). These results indicate that the inclusion of Al(OH)_3_ alone for NM2e immunization may significantly improve the protective efficacy of NM2e, whereas the inclusion of CpG only results in no such effect. Moreover, Al(OH)_3_ and CpG did not show a synergistic effect on mortality.

NM2e induced stronger immune response and higher protection efficacy than NP only in mice.

**Figure 7:**
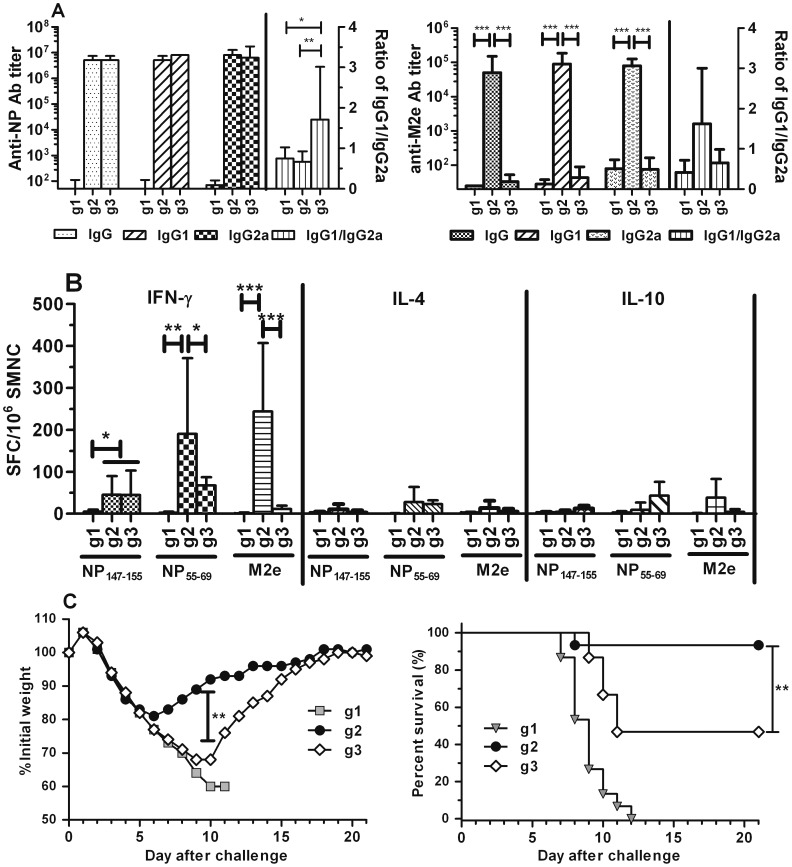
Comparison of the immunogenicity and protection efficacy induced by NM2e and NP. Mice were immunized intramuscularly with 10 µg of NM2e (g2)or NP protein (g3) formulated with Al(OH)_3_ according to the time schedule in Fig. 2. Mice immunized with adjuvant alone were used as negative controls (g1). Serum, SMNC was prepared from each mouse and analyzed at the indicated times as described in the Materials and Methods. A), Ab and subtypes against NP (left) and M2e (right) on day 38. Columns show geometric mean antibody titers, and bars indicate the 95% confidence interval in each group (*n* = 6 mice per experimental group). B), SMNCs secreting IFN-γ, IL-4, or IL-10 upon stimulation were detected by ELISPOT assay. Six mice in each treatment group were sacrificed on day 38. The numbers of SMNCs producing IFN-γ (left), IL-4 (middle), or IL-10 (right) after stimulation for 40 h with NP_147–155_, NP_55–69_, or M2e peptides are presented as spot-forming cells (SFCs)/10^6^ SMNCs. Columns show the average SFCs/10^6^ SMNCs, and bars indicate the standard deviation of each group. *, *p*≤0.05; **, *p*≤0.01; ***, *p*≤0.001 by one-way ANOVA. C), Protective efficacy of immunization with NM2e or NP formulated with Al(OH)_3_ in mice. Three mice group were challenged with 20 LD_50_ of influenza virus PR8 (*n = 15*). Mice were monitored daily to detect morbidity (left) and mortality (right). *, *p*≤0.05; **, *p*≤0.01; ***, *p*≤0.001.

To test whether NM2e was able to induce stronger immune response than NP, 10 µg of NM2e (group 2, g2) and NP protein (group 3, g3) formulated with Al(OH)_3_ were used to immune BALB/c mice three times respectively according to the time schedule in Fig. 2. Mice immunized with Al(OH)_3_ were treated as negative control (group 1, g1). Ab analysis result showed that after the third immunization NM2e formulated with Al(OH)_3_ induced comparable anti-NP IgG and IgG1, IgG2a with NP formulated with Al(OH)_3_ (Fig. 7A, left). Different from NP, NM2e did induce anti-M2e Ab (Fig. 7A, right). ELISPOT result showed NM2e induced comparable NP_147–155_ and NP_55–69_ specific cellular immune response with NP, more importantly it succeeded in inducing cellular response against M2e (Fig. 7B), whereas NP didn’t. Morbidity data showed NP immunized mice experienced significantly greater weight loss (*p*<0.01) than mice in group 2, the survival mice in group 3 began to recover their body weight on day 10, 4 days later than NM2e immunized mice (Fig. 7C, left). NP immunization did induce protective immunity, however the survival rate of mice group 3 was significantly lower than that in group 2 (*p*<0.01) (Fig. 7C, right).

### Protective Efficacy is Correlated with Humoral and Cellular Immune Responses

**Figure 8:**
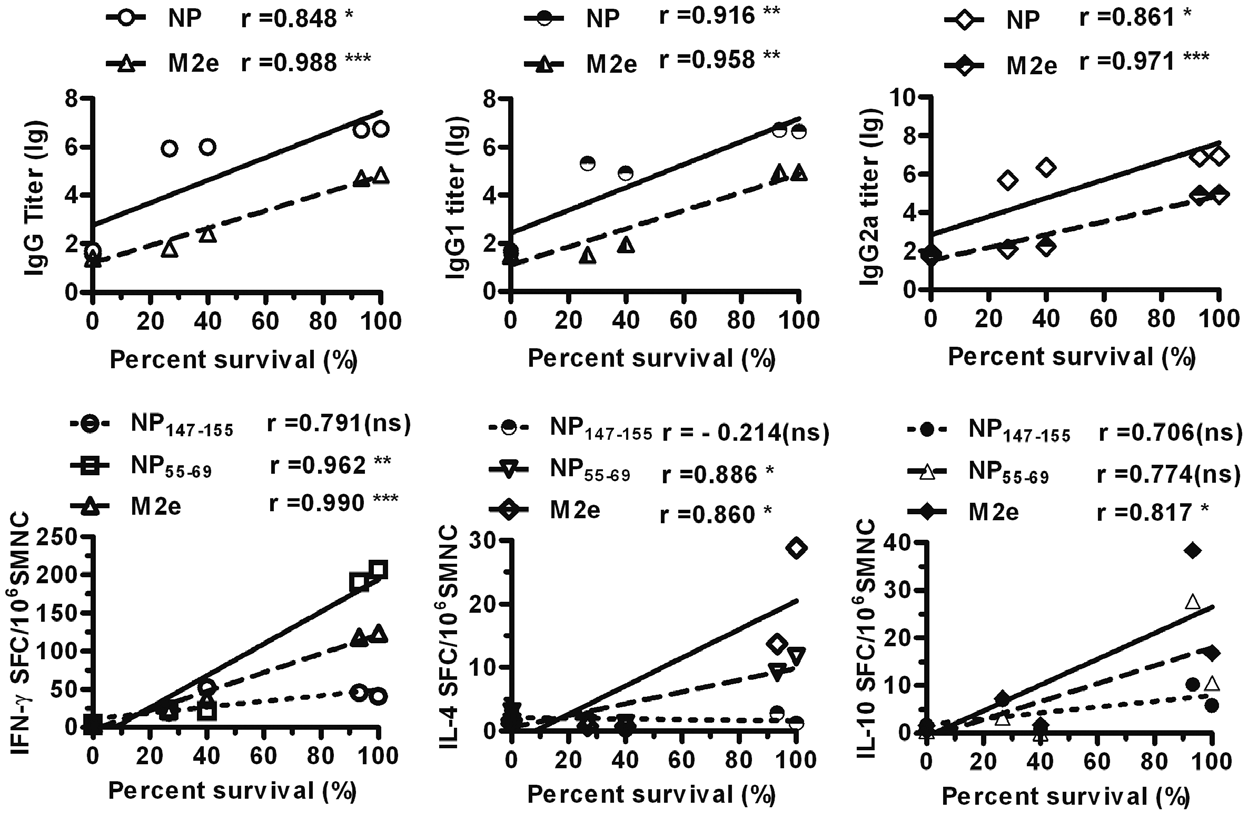
Correlations between survival percentage and immune responses in mice. A, Correlation analysis was conducted to determine the relationships of the survival percentage data from Fig. 6 with the NP-, M2e-specific IgG (left) ELISA data from Fig. 3, IgG1 (middle) and IgG2a (right) ELISA data in Fig. 4. Log conversion was performed for the murine serum antibody titers. B, Correlation analysis was conducted to determine the relationships of the survival percentage data in Fig. 6 with the IFN-γ- (left) IL-4- (middle), and IL-10-secreting (right) SMNCs stimulated with NP147-155, NP55-69, or M2e peptide pool based on the ELISPOT data in Fig. 5.

To better understand the relationship between protective efficiency and the humoral and cellular immune responses, correlation coefficients were analyzed. With respect to humoral immune responses, the survival percentage induced by NM2e immunization was highly related to NP- and M2e-specific total IgG, IgG1, and IgG2a antibody levels (Fig. 8A). Moreover, the survival percentage was markedly related to NP55-69- and M2e-specific cellular immune responses, but not to NP147-155-specific cellular immune responses (Fig. 8 B). Thus, the protective efficacy of NM2e immunization was related to both the humoral and cellular immune responses.

## Discussion

A universal influenza vaccine capable of inducing cross-protection among heterosubtypic influenza strains is critically needed to prevent seasonal and pandemic flu outbreaks. The highly conserved NP and M2 of influenza A virus have been used as target antigens in the development of universal influenza vaccines. In mice, previous studies have shown that gene- and/or vector-based NP+M2 vaccines induced strong humoral and cellular responses, and protected against lethal influenza virus challenges, the combination of NP and M2 is superior to the sole one when the immune response and protection efficacy were considered [25]–[31]. Here, we describe a vaccine based on a fusion protein of NP and M2e expressed in *E. coli*. In mice, the NM2e fusion protein elicited robust antibody responses and T cell responses against NP and M2e. More importantly, mice immunized with NM2e formulated with Al(OH)_3_ adjuvant were protected against lethal challenge with high doses of influenza A virus PR8 (20LD50) compared with the relatively low challenge dose (1 LD90 [12], [13] or 4 LD50 [11], [17]) in other studies with M2-based vaccines. It is noticeable here that NM2e did contain the character of both NP and M2e and induced stronger protective immune response than NP protein. The present results suggest that *E. coli*-expressed NM2e fusion protein is immunogenic in mice and may be a suitable candidate for a universal influenza vaccine.

It is necessary to understand the immune correlates of protection for new vaccine types. Here, NM2e immunization induced a substantial antibody response to M2e in mice, and an analysis showed that the protection was closely correlated with the anti-M2e antibody titer. Previous experiments have shown similar results [16], [43], [45]. Anti-M2e antibody is unable to neutralize the virus to prevent infectivity, but it is able to disrupt the viral life cycle and kill infected cells by the mechanism of antibody-dependent cell-mediated cytotoxicity (ADCC) [46]. M2e-specific IgG2a isotype has been proposed as an effective inducer of the ADCC response [47]. Jegerlehner *et al*. [46] suggested that IgG2a levels were correlated with protection against influenza infection in mice. However, Denis *et al*. [45] reported that low levels of anti-M2e IgG2a induced by PapMV-CP-M2e immunization did not efficiently protect mice against a challenge with 4 LD_50_ of influenza A virus; thus, they considered that anti-M2e IgG1 may also play a role in M2e-mediated protection. Our results demonstrated that protection was highly correlated with not only IgG2a but also IgG1. The M2e peptide contains an MHC class II-restricted epitope [11], and studies have documented that influenza-specific CD4+ T cells are involved in immune protection [11], [48]. In the present study, M2e-specific IFN-γ-, IL-4-, and IL-10-secreting SMNCs (mainly CD4+ T cells; data not shown) were significantly correlated with protection. It was reported that following influenza infection, antigen-presenting cells secrete IL-10, which contributes to the differentiation of Th0 cells into Th2 cells; subsequently, Th2 cells secrete IL-4, IL-5, and IL-6, which help to preferentially drive IgG1, IgA, and IgE antibody production by antibody-secreting plasma cells [49]. Th1 cells secrete IFN-γ, which helps to produce IgG2a antibodies [50]. Thus, our data indicate that both M2e-specific antibodies and CD4+ T cells contribute to the protection induced by NM2e protein.

Several studies have reported that NP plays a role in the elimination of influenza virus-infected cells via specific CD8+ killer T cells [22], [26], [27], [51]. However, recent studies have suggested that antibodies against NP are necessary for NP immunization to confer protection and that NP-immune serum can transfer protection [36], [52], [53]. Although NP-specific IgG antibodies had no effect on neutralization and failed to block viral infection of cells, antibodies to NP may nevertheless provide an unexpected yet important mechanism of protection against influenza [11], [18]. In the present study, protection against virus challenge was closely correlated with the presence of anti-NP antibodies, including IgG1 and IgG2a isotypes. Furthermore, the protective effect was significantly correlated with SMNCs specific for NP55–69 (H-2^d^-restricted Th epitope), but not SMNCs specific for NP147–155 (H-2^d^-restricted CTL epitope). It is not surprising that NP-specific CD8+ T cells were not elicited in response to NM2e immunization because although gene- and vector-based NP vaccines easily induce CD8+ T cells, protein vaccines might not, even with CpG as adjuvant. Further research is needed to identify a suitable adjuvant that favors the induction of CD8+ T cells in response to NM2e protein vaccination. Meanwhile, it is essential to identify the mechanism of action of NM2e immunity and to establish *in vitro* assays for measuring immunity.

Adjuvant is required for protein subunit vaccines to elicit effective and long-lasting immune protection [38]. In this study, inclusion of the Al(OH)_3_ adjuvant, which is widely and safely used in human vaccines [54], enhanced both humoral and cellular immune responses elicited by NM2e. However, it should be noted that alum boosts mainly the Th2 immune response, while it inhibits the Th1 and CTL response elicited by many antigens [38], [54], which was also shown in this study. Different from the immune mechanism of alum adjuvant, CpG efficiently promotes Th1 and CTL responses [55]–[57]. In the present study, inclusion of CpG alone considerably enhanced the humoral immune response elicited by NM2e, however it did not enhance the CTL response markedly. The inclusion of CpG with NM2e improved the anti-M2e IgG1 titer, but not the IgG2a titer (Fig. 4, right), which was different from the NP-specific IgG1/IgG2a pattern (anti-NP IgG1 titer decreased and anti-NP IgG2a titer increased). McCluskie MJ et al [58] once reported CpG together with the *E. coli* heat-labile enterotoxin (LT) strengthened the humoral response against some antigen, while others not, so adjuvanticity may have also depended on the particular antigen. NP is one of the internal protein of influenza virus, while M2e is the extracellular domain of the membrane protein 2, thus we infer CpG showed different adjuvanticity when two different antigens were used. In addition, Shim B-S [37] once prepared the construct expressing three tandem copies of M2e conjugated to C-terminus sequence of M2 protein (3M2eC), their research showed that immunization with 3M2eC induced predominantly IgG1 as compared to IgG2a subclass, similar to the result in this study.

CpG was expected to complement the immune effect induced by Al(OH)_3_ when used together in the formulation of NM2e protein. However, CpG plus Al(OH)_3_ did not show a clear synergistic effect on the immunogenicity of NM2e. It is possible that remnant endotoxin (about 2000 EU/mg) in the NM2e formulation expressed in *E. coli* might have interfered with the effect of CpG. Our recent data indicated that once remnant endotoxin was removed further from the NM2e protein formulation, CpG and Al(OH)_3_ used together did show a clear synergistic effect on the immune response (Fig. S1) and protective efficacy (Fig. S2) of NM2e, and inclusion of Al(OH)_3_ alone still markedly improved the immune efficacy of the NM2e vaccine.

In conclusion, we have described a potential universal influenza vaccine that provides cross-protective immunity against influenza A virus PR8 in mice. The vaccine is based on the recombinant fusion protein NM2e expressed in *E. coli*, which consists of the 23-amino acid external domain of M2 protein attached to the C-terminus of NP. When administered with Al(OH)_3_ as adjuvant, NM2e provided almost complete protection against a lethal-dose challenge of A/PR8 in mice. The combination of Al(OH)_3_ and NM2e offers promising prospects for further vaccine development.

## Supporting Information

Figure S1 The study of the immunogenicity of NM2e in pre-production in mice. The pre-production fermentation and purification of NM2e were finished in SINOVAC BIOTECH CO.,LTD., Beijing, China. The concentration of endotoxin in NM2e protein was decreased to 250 EU/mg after the purification. Mice were immunized with 10 µg of NM2e (G3), 10 µg of NM2e formulated with Al(OH)_3_ (G5), 10 µg of NM2e formulated with Al(OH)_3 _plus CpG (G6) according to the procedure in Fig. 2. Mice immunized with NS (G1), Al(OH)_3 _plus CpG (G2) were treated as negative controls. A), Ab and subtypes against NP (left) and M2e (right) on day 38 were analyzed by ELISA. Columns show geometric mean antibody titers, and bars indicate the 95% confidence interval in each group (*n* = 6 mice per experimental group). B), SMNCs secreting IFN-γ, IL-4, or IL-10 upon stimulation were detected by ELISPOT assay. Six mice in each treatment group were sacrificed on day 38. The numbers of SMNCs producing IFN-γ (left), IL-4 (middle), or IL-10 (right) after stimulation for 40 h with NP_147–155_, NP_55–69_, or M2e peptides are presented as spot-forming cells (SFCs)/10^6^ SMNCs. Columns show the average SFCs/10^6^ SMNCs, and bars indicate the standard deviation of each group. *, *p*≤0.05; **, *p*≤0.01; ***, *p*≤0.001 by one-way ANOVA.
(TIF)

Figure S2 Protective efficacy of immunization with the formulation of NM2e in pre-production in mice. Immunized mice (*n = 15*) were challenged with 30 LD_50_ of influenza virus A/Brisbane/59/2007(H1N1)-like (MA) on day 38, then they were monitored daily to detect morbidity (left) and mortality (right). *, *p*≤0.05; **, *p*≤0.01; ***, *p*≤0.001.
(TIF)
