# Supplementary figures and images for: Robust Immunity and Heterologous Protection against Influenza in Mice Elicited by a Novel Recombinant NP-M2e Fusion Protein Expressed in E. coli
Source: PLoS One. 2012 Dec 21;7(12):e52488. doi: 10.1371/journal.pone.0052488 (PMC3528677; doi:10.1371/journal.pone.0052488)

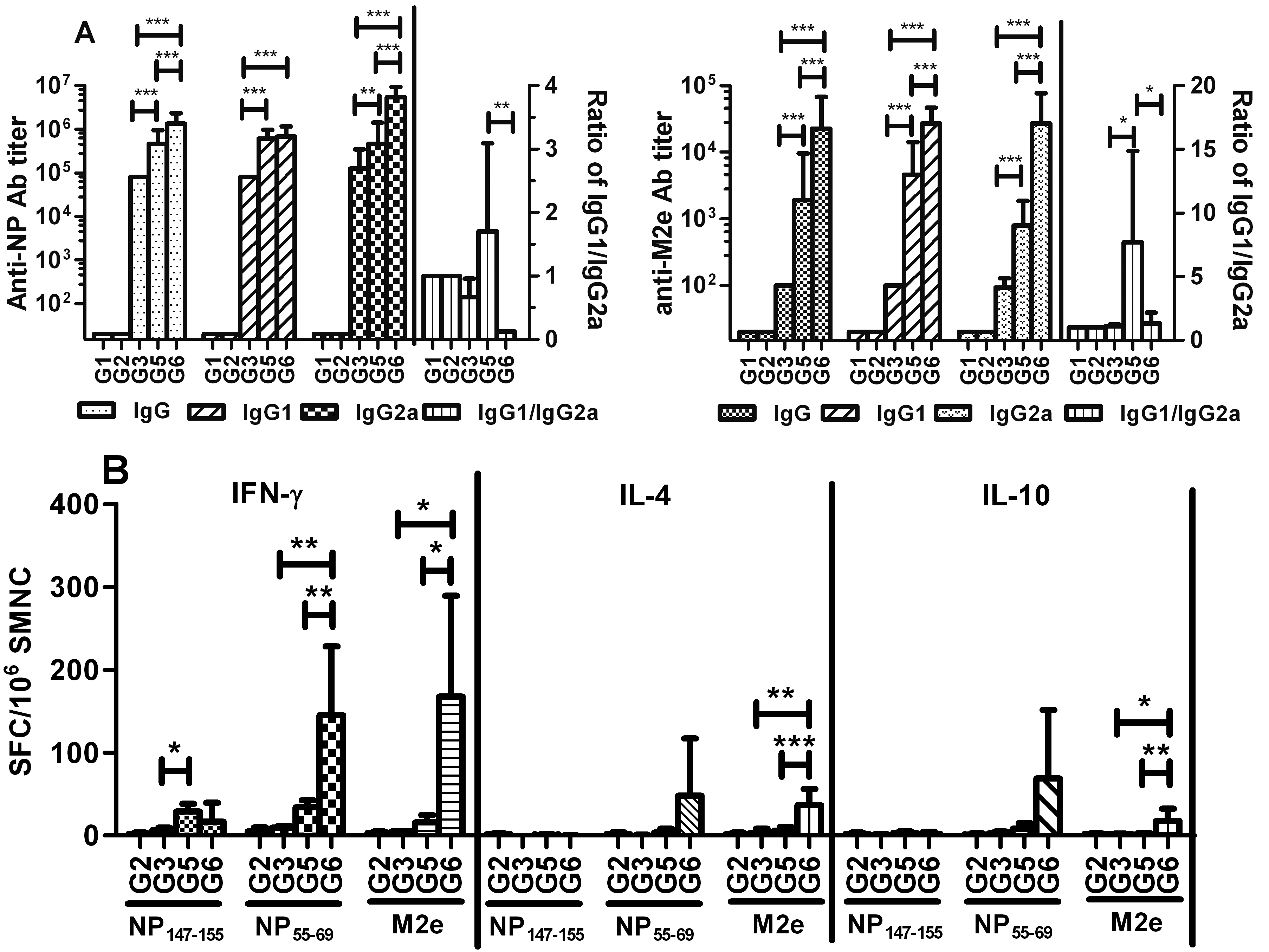

Supplement: Figure S1 — The study of the immunogenicity of NM2e in pre-production in mice. The pre-production fermentation and purification of NM2e were finished in SINOVAC BIOTECH CO.,LTD., Beijing, China. The concentration of endotoxin in NM2e protein was decreased to 250 EU/mg after the purification. Mice were immunized with 10 µg of NM2e (G3), 10 µg of NM2e formulated with Al(OH)3 (G5), 10 µg of NM2e formulated with Al(OH)3 plus CpG (G6) according to the procedure in Fig. 2. Mice immunized with NS (G1), Al(OH)3 plus CpG (G2) were treated as negative controls. A), Ab and subtypes against NP (left) and M2e (right) on day 38 were analyzed by ELISA. Columns show geometric mean antibody titers, and bars indicate the 95% confidence interval in each group (n = 6 mice per experimental group). B), SMNCs secreting IFN-γ, IL-4, or IL-10 upon stimulation were detected by ELISPOT assay. Six mice in each treatment group were sacrificed on day 38. The numbers of SMNCs producing IFN-γ (left), IL-4 (middle), or IL-10 (right) after stimulation for 40 h with NP147–155, NP55–69, or M2e peptides are presented as spot-forming cells (SFCs)/106 SMNCs. Columns show the average SFCs/106 SMNCs, and bars indicate the standard deviation of each group. *, p≤0.05; **, p≤0.01; ***, p≤0.001 by one-way ANOVA. (TIF) [file pone.0052488.s001.tif]

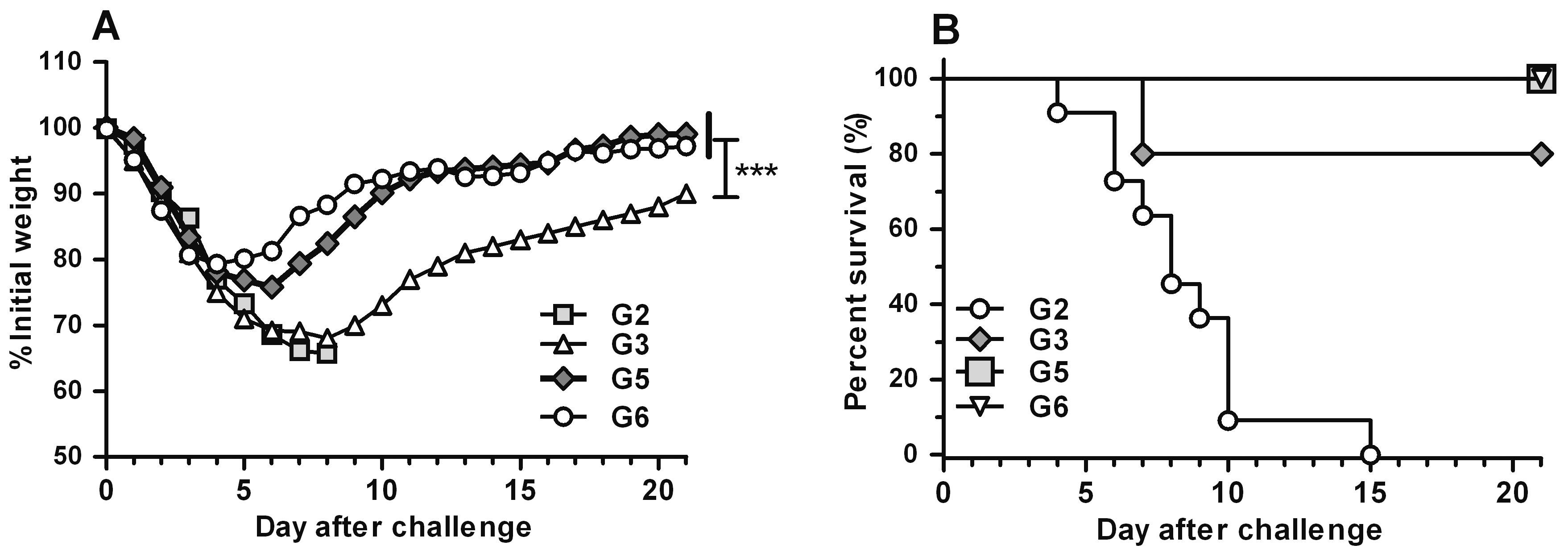

Supplement: Figure S2 — Protective efficacy of immunization with the formulation of NM2e in pre-production in mice. Immunized mice (n = 15) were challenged with 30 LD50 of influenza virus A/Brisbane/59/2007(H1N1)-like (MA) on day 38, then they were monitored daily to detect morbidity (left) and mortality (right). *, p≤0.05; **, p≤0.01; ***, p≤0.001. (TIF) [file pone.0052488.s002.tif]
